# Genetic Engineering of Bacteriophages Against Infectious Diseases

**DOI:** 10.3389/fmicb.2019.00954

**Published:** 2019-05-03

**Authors:** Yibao Chen, Himanshu Batra, Junhua Dong, Cen Chen, Venigalla B. Rao, Pan Tao

**Affiliations:** ^1^ College of Veterinary Medicine, Huazhong Agricultural University, Wuhan, China; ^2^ The Cooperative Innovation Center for Sustainable Pig Production, Huazhong Agricultural University, Wuhan, China; ^3^ Department of Biology, The Catholic University of America, Washington, DC, United States

**Keywords:** bacteriophages, genome engineering, vaccine platform, phage therapy, infectious disease

## Abstract

Bacteriophages (phages) are the most abundant and widely distributed organisms on Earth, constituting a virtually unlimited resource to explore the development of biomedical therapies. The therapeutic use of phages to treat bacterial infections (“phage therapy”) was conceived by Felix d’Herelle nearly a century ago. However, its power has been realized only recently, largely due to the emergence of multi-antibiotic resistant bacterial pathogens. Progress in technologies, such as high-throughput sequencing, genome editing, and synthetic biology, further opened doors to explore this vast treasure trove. Here, we review some of the emerging themes on the use of phages against infectious diseases. In addition to phage therapy, phages have also been developed as vaccine platforms to deliver antigens as part of virus-like nanoparticles that can stimulate immune responses and prevent pathogen infections. Phage engineering promises to generate phage variants with unique properties for prophylactic and therapeutic applications. These approaches have created momentum to accelerate basic as well as translational phage research and potential development of therapeutics in the near future.

## Introduction

Bacteriophages (phages), discovered in the early 20th century independently by Frederick Twort and Felix d’Herelle, are the most abundant organisms on earth with up to 2.5 × 10^8^ phages per ml in natural waters (Bergh et al., 1989). It is well accepted that phages specifically infect bacteria and, therefore, were considered for the development of natural approaches to treat bacterial infections since their discovery (Wittebole et al., 2014; Salmond and Fineran, 2015). However, due to the discovery of antibiotics that provided greater breadth and potency, phage therapy lagged behind although research continued in some Eastern European countries (Chanishvili, 2012, 2016; Wittebole et al., 2014). Therefore, in the following several decades, phages were mainly used as model organisms to explore the basic mechanisms of life and led to the birth of modern molecular biology. One classical example is the demonstration of a central biological question in the early 20th century, the nature of a gene, by “Hershey-Chase experiment” (also called “Waring blender experiment”) (Salmond and Fineran, 2015). This elegant experiment demonstrated that DNA, not protein, is the genetic material of T2 phage.

Recently, the emergence of multi-antibiotic resistant bacterial pathogens and the low rate of new antibiotic discovery brought new urgency to develop phage-based therapies (Lu and Koeris, 2011; Viertel et al., 2014; Domingo-Calap and Delgado-Martinez, 2018). A striking example is the recent San Diego patient who was infected by multi-drug resistant *Acinetobacter baumannii* stain during travelling to Egypt. The patient went into a coma for nearly 2 months but awoke 2 days after intravenous injection of a phage cocktail that lyses this bacterium and finally completely recovered (Schooley et al., 2017). With recent advances, particularly the genome engineering (Martel and Moineau, 2014; Ando et al., 2015; Lemay et al., 2017; Tao et al., 2017b; Kilcher et al., 2018), the applications of phages have greatly expanded. In addition to its use in antibacterial therapy, phages were used in synthetic biology (Lemire et al., 2018), material science (Cao et al., 2016), and biomedical fields (Cao et al., 2018; Tao et al., 2018c). Considering the abundance and diversity, there is vast potential to engineer phages for different applications. In this review, we will focus on the applications of phages in infectious disease, in particular, vaccine development and phage therapy. We will discuss the phage engineering strategies and how these can equip the phages with the ability to advance the vaccine and phage therapy fields.

## Phage Genome Engineering

### Traditional Homologous Recombination-Based Techniques

**Figure 1:**
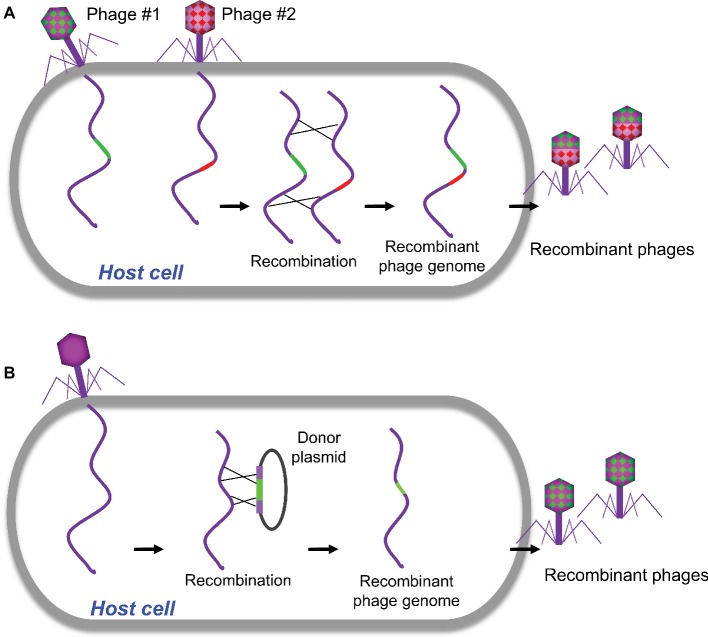
Traditional homologous recombination-based phage engineering. **(A)** Classical “phage cross” to generate mutant phages with two parent phages. **(B)** Phage mutants generated by homologous recombination between the plasmid and wild-type phage genome.

Homologous recombination refers to the exchange of nucleotide sequences between two DNA molecules, which share similar or identical sequences. It is a naturally occurring biological event and was employed in the first-generation strategy for engineering phage genomes. This classical genetic strategy (called phage crosses) was used as a standard way to generate a mutant phage with specific phenotypes by either combining or separating mutations from two parental phages (Figure 1A; Karam et al., 1994). Host cells were co-infected with two parental phages, which at least have two selective markers (or phenotypes). The homologous recombination will occur between parental phage genomes. The progeny phages were then screened for the desired phenotype(s), and the recombinants with appropriate phenotypes were purified for further analysis. Obviously, this approach was mainly used to exchange or combine the phenotypes of parental phages and was unable to do specific modification to the targeted site in phage genome, which limits the use of the method.

Homologous recombination between the plasmid and phage genome was then developed to generate recombinant phages with gene replacements, deletions, or insertions (Sarkis et al., 1995; Rao and Mitchell, 2001; Oda et al., 2004; Tanji et al., 2004; Namura et al., 2008). In a standard procedure, the plasmid containing a designed mutation flanked by homologous sequences of phage is constructed and transformed into host bacteria, which is then infected with the phage to be engineered (Figure 1B). The resulted recombinant phages containing the desired mutations were then screened. Although higher recombination rates up to 5 × 10^−3^ were reported for the some phages (Oda et al., 2004), overall the frequencies of recombination are quite low (Sarkis et al., 1995; Tanji et al., 2004). Therefore, this classical genetic strategy is tedious and time-consuming to find the desired recombinants unless there is a selection strategy for the recombinant phage.

### Bacteriophage Recombineering of Electroporated DNA (BRED)

Recombineering is also a homologous recombination-based technique but exploits a phage-encoded recombination system such as Red system of phage lambda and RecE/RecT system of Rac prophage to enhance the frequency of homologous recombination (Poteete, 2001; Murphy, 2012; Nafissi and Slavcev, 2014). Red system is a well-studied phage recombination system composing the *gam* (γ), *exo* (α), and *bet* (β) genes. Gam inhibits *E. coli* RecBCD exonuclease complex to prevent degradation of the liner dsDNA substrate (Poteete, 2001). Exo targets double-stranded DNA (dsDNA) ends to degrade one strand of DNA in a 5′ to 3′ manner to generate a single-stranded DNA (ssDNA) substrate. Beta is an ssDNA-binding protein that anneals the ssDNA substrate to its recombination target in phage genome.

Similar to the plasmid-based homologous recombination mentioned above, BRED requires co-electroporation of the phage DNA template and donor DNA into bacterial cells expressing proteins such as RecE/RecT-like proteins *via* either plasmid or chromosomally inserted genes to promote homologous recombination (Marinelli et al., 2008, 2012; Thomason et al., 2009). The donor DNA contains the desired mutations flanked by homologous sequences of phage to be engineered, which lead to the homologous recombination occurring between phage genome and donor DNA. It was suggested that recombination happens only after phage genome replication has begun (Marinelli et al., 2008). Therefore, wild-type phages will be also recovered along with the mutant phages, and the generated plaques contain a mixture of wild-type and mutant phages. When using this method to engineer mycobacteriophage Giles, the mutant-containing plaques can be recovered at an efficiency of 3.4–22.2% (Marinelli et al., 2008). However, after initial PCR screening of plaques containing mutants, further purification are needed to isolate a homogenous phage mutant (Marinelli et al., 2008). This method was first developed for mycobacteriophage and was later on adapted to many other phages to construct gene deletions, replacements, and heterologous gene insertions (Thomason et al., 2009; Marinelli et al., 2012). However, it highly relies on co-transformation of phage DNA and donor DNA into the same cell, which is generally low. Therefore, this method is especially difficult to use in Gram-positive bacteria that exhibit low transformation efficiencies. Instead of co-transformation of phage DNA and donor DNA, bacteria that contain phage-encoded recombination system can be transformed only with donor DNA (Oppenheim et al., 2004; Pan et al., 2017). The phage mutants will be then generated by infecting bacterial cells with WT phage. This will overcome the transformation limit of BRED to some extent. However, high background of WT phage is expected, thus extensive screening of recombinants is required.

### CRISPR-Cas-Based Phage Engineering

**Figure 2:**
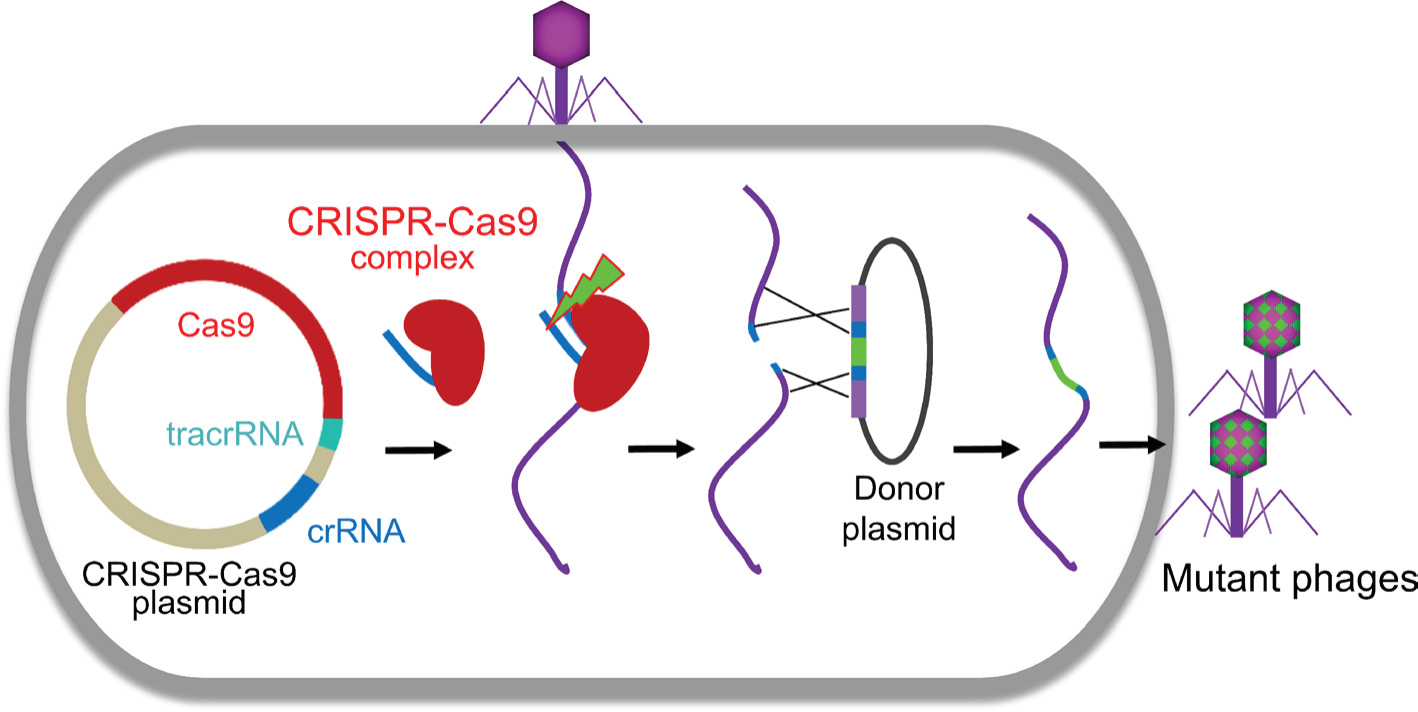
CRISPR-Cas-based phage engineering. The formed CRISPR-Cas9 complex specifically binds to the target site in the phage genome and creates a double-strand DNA break during phage infection. The mutations were introduced into the donor plasmid. The DNA break can be repaired by recombination with the donor to generate mutants of interest.

Clustered regularly interspaced short palindromic repeats (CRISPR)-Cas system, firstly discovered at the end of last century, is an immune system of prokaryote to counter the invasions. Recently, it was adapted for genome engineering in many organisms including phages (Figure 2; Box et al., 2016; Bari et al., 2017; Tao et al., 2017b; Hatoum-Aslan, 2018; Hupfeld et al., 2018; Knott and Doudna, 2018; Schilling et al., 2018; Shen et al., 2018). The effector complexes of CRISPR-Cas system contain two main components, Cas proteins and CRISPR RNA (crRNA). The effector complexes specifically bind to their target sequences mediated by the crRNA with a region complementary to the target DNA where Cas protein cleaves the DNA and creates a double-strand DNA break (Shmakov et al., 2017; Knott and Doudna, 2018). The CRISPR-Cas system can be classified into six types and further cataloged into two broad classes (Class 1 or 2) based on phylogeny and activity mechanisms (Koonin et al., 2017). Class 1 systems, including types I, III, and IV, employ effector complexes containing multiple Cas proteins, while class 2 systems, including types II, V, and VI, employ effector complexes containing a single Cas protein to cleave the target DNAs (Koonin et al., 2017).

CRISPR-Cas system was first applied to phage genome editing in 2014 to select a T7 phage mutant with a deletion of a nonessential gene, *gene1.7* (Kiro et al., 2014). In this study, CRISPR-Cas system was used as a screening tool to eliminate the WT phage from the recombinants. This plasmid-based type I CRISPR-Cas system, which was targeted to *gene 1.7*, was able to cleave WT genome and eliminate WT T7 phages. In contrast, the mutant phages lacking *gene 1.7* were resistant to Cas9 complex and could propagate normally (Kiro et al., 2014). Later on, type I CRISPR-Cas system from *Vibrio cholerae* was identified and used for engineering of *V. cholerae* lytic phage (Box et al., 2016). In this system, both donor DNA and CRISPR-Cas components were assembled in a single plasmid. Propagation of phages on *V. cholerae* harboring this plasmid led to the cleavage of phage genome by CRISPR-Cas, which was repaired by homologous recombination with the donor DNA and resulted in recombinant phages with deletion or insertion mutations (Box et al., 2016).

Although the first type II CRISPR-Cas system used for phage editing was from *Streptococcus thermophiles* (Martel and Moineau, 2014), *Streptococcus pyogenes* CRISPR-Cas is most often used for phage genome engineering (Lemay et al., 2017; Tao et al., 2017b; Schilling et al., 2018; Shen et al., 2018). Recently, CRISPR-Cas of *Listeria monocytogenes* was also identified and used to develop an effective engineering platform for *Listeria* phages (Hupfeld et al., 2018). Usually, all three components of CRISPR-Cas system, Cas9, crRNA, and trans-activating crRNA (tracrRNA), were cloned into a single plasmid. The crRNA and tracrRNA could be either expressed separately (Lemay et al., 2017; Tao et al., 2017b) or as a single fusion RNA (Schilling et al., 2018). After transform into host cells, all the components were expressed and formed a CRISPR-Cas9 complex, which will specifically bind to the target site in the phage genome and creates a double-strand DNA break during phage infection (Figure 2). Due to the absence or low efficiency of non-homologous end joining (NHEJ) repairing systems in bacteria (Brissett and Doherty, 2009), the cleavage of CRISPR-Cas9 complex is usually lethal to the phage (Tao et al., 2017b, 2018b). When the homologous donor is provided, the DNA break can be repaired by recombination with the donor to generate mutants of interest. We found that the *S. pyogenes* Cas9 complex can even efficiently cleave T4 phage genome, which is highly resistant to most restriction endonucleases due to the covalent modifications (5-hydroxymethylation and glucosylation) to its cytosines (Tao et al., 2017b, 2018b). However, the cleavage efficacy of CRISPR-Cas9 complex depends on the selected crRNA (Tao et al., 2017b, 2018b). When the crRNA targeting site (protospacer sequence) in the phage genome is highly vulnerable to cleavage by Cas9 complex (high restriction spacer), only the recombinant phages can survive. Therefore, all resultant progeny phages are recombinant mutants. However, if the protospacer is poorly cleaved (low restriction spacer) or an overdose of parental phages were used for infection, it could lead to error-prone repair and incorporation of random mutations in the protospacer sequence resulting in escape from CRISPR-Cas cleavage (Martel and Moineau, 2014; Tao et al., 2018b). This was reported in both types I and II CRISPR-Cas systems (Barrangou et al., 2007; Fineran et al., 2014).

The type III CRISPR-Cas system was also used for engineering virulent staphylococcal phages. This method utilizes the native endogenous CRISPR-Cas10 system of *Staphylococcus epidermidis* but supplemented with the crRNA transcribed from an exogenous plasmid (Bari et al., 2017). This CRISPR-Cas10 system has high cleavage efficacy and affords *S. epidermidis* complete protection against a high dose of staphylococcal phages (Andhra and ISP) infection when coding strand is targeted. The donor DNA was also cloned into the same plasmid expressing crRNA. Infection *S. epidermidis* containing this plasmid with staphylococcal phages was able to generate progenies. Strikingly, all the tested progeny phages acquired the desired mutations (Bari et al., 2017).

### Rebooting Phages Using Assembled Phage Genomic DNA

**Figure 3:**
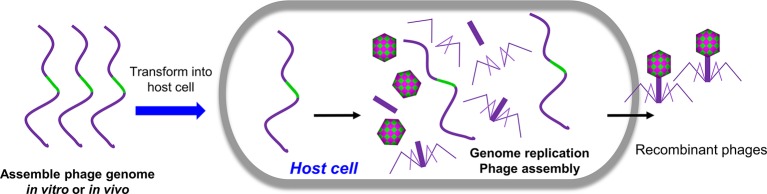
Rebooting phages using assembled phage genomic DNA. The phage genome DNA with desired mutations was assembled *in vivo* or *in vitro* and was transformed into host cells. The replication, transcription, and translation of genomic DNA in the host cells will lead to the assembly of infectious phages.

In principle, all the methods mentioned above are based on the homologous recombination. Alternatively, engineered phages can be directly generated by transforming the host cells with naked full-length phage genomic DNA containing the desired mutations (Figure 3). Replication, transcription, and translation of genomic DNA in the host cells will lead to the assembly of infectious phages. For phages with small genome such as phiX174 (5,386 bp), the genomic DNA can be assembled *in vitro via* polymerase cycling assembly (PCA) using synthetic oligonucleotides that span the whole genome with overlap sequences (Smith et al., 2003; Mamedov et al., 2007). For phages with larger genomes such as T7 (39,937 bp), the genomic DNA can be assembled *in vitro* through ligation of individual genome fragments cut with specific restriction enzymes (Chan et al., 2005). Alternatively, the full-length genomic DNA can be assembled with overlapping genome fragments *in vivo* through transformation-associated recombination (TAR), which exploits a high level of homologous recombination in the yeast (Jaschke et al., 2012; Ando et al., 2015). The TAR approach has been used to assemble large DNA up to 300 kb in length (Hou et al., 2016; Shang et al., 2017). The overlapping viral fragments were amplified from the genome by PCR, and each adjacent fragment has a homologous sequence overhang. The first and last phage fragments contain homology sequences with yeast vector. When transformed into yeast cells, the fragments were recombined to form a complete phage genome in a yeast vector. The DNA was then extracted from yeast and transformed into host cells to generate phages. Mutations can be introduced into any of the fragment(s) to generate desired phage mutants.

Gram-positive bacteria usually exhibit low transformation efficiencies. A recent study indicated that it is also possible to efficiently reboot phages of Gram-positive bacteria using L-form bacteria as rebooting compartments (Kilcher et al., 2018). L-form bacteria are cell wall-deficient bacteria, which, unlike its parent cells, have the ability to take up large DNA such as phage genome DNA. It was shown that L-form *Listeria* can be employed not only for rebooting of *Listeria* phages but also enable cross-genus rebooting of *Bacillus* and *Staphylococcus* phages (Kilcher et al., 2018).

## Applications of Phages in Infectious Disease

Infectious diseases can be treated before (prophylaxis) or after (therapy) infection, where phages can contribute at both levels to treat bacterial infections (Debarbieux et al., 2010; Chanishvili, 2016; Tao et al., 2018c). Phages have been used to eliminate bacterial pathogens since their discovery last century. Recent studies also indicated their high potential to be developed as vaccine platforms, which can be used to prevent both bacterial and viral pathogens. Here, we discuss the characters that make phages good candidates against infectious diseases either as vaccine platform or as phage therapy. Phage engineering technologies allow generate variants with unique properties and help minimum the features that might hamper the applications of phage for prophylactic and therapeutic applications.

### Vaccines

The immune system formed during long-term evolution can efficiently recognize and eliminate pathogens, such as viruses, through producing pathogen-specific immune response. From this point of view, many viral features, such as size, geometry, highly ordered and repeat structure, and multivalent display, which are critical for eliciting immune response, can be used to guide vaccine design (Bachmann and Jennings, 2010; Zepp, 2010). Phages are natural viruses that only infect bacteria, but have similar properties as mammalian viruses, and therefore can efficiently stimulate immune response (Jonczyk-Matysiak et al., 2017). Therefore, they have the high potentiality to be used as scaffolds to develop broadly applicable vaccine platforms (Fehr et al., 1998; Tissot et al., 2010; Nicastro et al., 2014; Henry et al., 2015; Fu and Li, 2016; Tao et al., 2018c). So far, many efforts have been focused on this topic, and many vaccine platforms have been developed using different phages, such as filamentous phages (Henry et al., 2015), phages λ (Nicastro et al., 2014), T4 (Tao et al., 2013a,b), T7 (Danner and Belasco, 2001), MS2 (Fu and Li, 2016), Qβ (Fehr et al., 1998), and others (Tissot et al., 2010).

The basic principle using phage as antigen delivery vehicles involves assembly of the pathogen antigen on phage capsid either *in vivo* or *in vitro* to form a virus-like particle (VLP) through fusion of antigen to a virus capsid protein. The antigens, therefore, are presented on capsid surface in a highly ordered and repetitive format, which is critical for activation of innate immune systems (Shepardson et al., 2017). For *in vivo* assembly, the antigen gene has to be inserted into phage genome to form a fusion gene of antigen and capsid protein. For phages with small genomes, it is relatively easy to generate such mutant phages. However, for complex phages such as T4, this could be labor intensive and time consuming. Thanks to recent progresses on genome engineering technology, such as the CRISPR-Cas system, there is now no fundamental obstacles to engineering such phages.

Due to viral characters and its CpG (a ligand for Toll-like receptor 9)-containing genome DNA, phages are able to stimulate innate immune and therefore potentially act as a natural adjuvant (Zimecki et al., 2003; Kaur et al., 2012; Sartorius et al., 2015). Therefore, it might be that the display of antigens on phages links the antigen to a self-adjuvanting vaccine delivery system, which might elicit robust immune responses without any external adjuvants. Indeed, our studies indicated that antigens assembled on T4 capsid elicited stronger immune responses compared to their soluble counterparts (Tao et al., 2013a, 2018a). Furthermore, loading antigens on a self-adjuvanting delivery system allows the simultaneous delivery of both the components to the same immune cells such as antigen-presenting cells (APCs), which could significantly enhance the immune responses. For example, significant higher level of antigen-specific IgG antibodies was induced when displayed on phage Qβ VLPs packaged with CpG than that of a simple mixture of antigen and CpG-packaged Qβ VLPs (Gomes et al., 2017).

Furthermore, presenting antigens on phage capsid surface in a highly ordered and repetitive format facilitates the binding of IgM to antigen epitope. For example, phage Qβ capsid could bind to natural IgM and fix complement component 1q and therefore efficiently deposit on follicular dendritic cells (FDCs) (Link et al., 2012), which is essential for the selection of B cell during germinal center reactions (Baschong et al., 2003; Hinton et al., 2008). However, the soluble capsid protein failed to activate this humoral innate immune response and cannot efficiently deposit on FDCs (Link et al., 2012).

Another advantage of the phage VLPs is that they are particulate antigens with highly localized epitope density on the surface, which can be presented by both class I and class II major histocompatibility complex (MHC) and therefore activate both CD4+ and CD8+ T cells (Mantegazza et al., 2013). Assembly of antigen proteins on phage capsids forms a VLP vaccine, therefore, efficiently activating CD4+ and CD8+ T cells. Indeed, our study showed that HIV-1 p24 antigens assembled on T4 capsid were able to induce p24-specific CD8+ T cells in immunized mice. In contrast, soluble p24 protein elicited significantly lower or no p24-specific CD8+ T cells (Sathaliyawala et al., 2006). Similarly, F1mutV antigen of *Yersinia pestis* activated both type 1 and type 2 helper T cells in mice when assembled on T4 capsid, whereas soluble F1mutV antigen mainly activated type 2 helper T cells (Tao et al., 2013a, 2018a).

Since phage capsids are usually composed of hundreds of capsid protein(s) (Nicastro et al., 2014; Chen et al., 2017; Tao et al., 2018c), assembly of antigens on a phage capsid will result in highly localized epitope density, which was seen in most of the licensed viral vaccines (Cheng, 2016). The highly localized epitope density was suggested to facilitate B cell activation through promoting cross-linking of the B cell receptors to antigens (Bachmann et al., 1993; Bachmann and Zinkernagel, 1996). Indeed, the study using Qβ phage capsid indicated that a high density of a model peptide (D2) induced higher titers of D2-specific IgG than medium or low density (Jegerlehner et al., 2002). Targeting of antigens to immune cells is considered to be one of the promising strategies to enhance vaccine efficacy (Kastenmuller et al., 2014; Macri et al., 2016). The dendritic cells (DCs) are the most popular target immune cells due to its key roles in connecting innate and adaptive immune response (Steinman and Banchereau, 2007). Since there is no mammalian tropism, phages can be engineered to target to DCs through displaying a DC-specific targeting molecule and, therefore, enhancing the immune response against the delivered antigens. For instance, the phage fd was engineered to display a single-chain variable fragment (scFV) of antibody against a DC-specific receptor (DEC-205) and an ovalbumin peptide through pIII and pVIII capsid protein, respectively (Sartorius et al., 2015). When injected into mice, the resulted phages induced higher level antibody titers compared to the phages that only display ovalbumin peptide but lack targeting molecule (Sartorius et al., 2011). Most phages have more than one structural protein that can be used to display and, therefore, are able to link the antigen and the DC-targeting molecule to the same VLP. For example, we have shown that the two nonessential capsid proteins, Hoc and Soc, of phage T4 can be used to simultaneously display two different foreign proteins (Li et al., 2007; Shivachandra et al., 2007; Tao et al., 2017a), one an antigen and another a DC-targeting molecule (e.g., a monoclonal antibody against DEC-205) (Tao et al., 2013b).

Although phages have many advantages as described above, to date, no vaccines employing a phage platform have yet been commercialized. Several phage platform-based vaccine candidates are undergoing clinical trials (Low et al., 2014; Huang et al., 2017), but most of these are still restricted to basic research. One reason could be that most of phages are not able to display the antigen in a high density as a full antigen, which is required for inducing high titers of conformation-specific neutralizing antibodies (Li et al., 2014). Additionally, pathogens can easily mutate certain key amino acids in the epitopes, making peptide vaccines based on one or a few epitopes less effective. However, recent progress on T4 phage platform showed that it is possible to display full-length antigen at high density. For instance, up to 360 copies of 83 kDa protective antigen (PA) (Li et al., 2007; Tao et al., 2018a), 350 copies of 90 kDa lethal factor (LF) (Li et al., 2007), 650 copies of 66 kDa plague F1mutV (Tao et al., 2013a, 2018a), and 200 copies of tetrameric 129 kDa β-galactosidase (Tao et al., 2013b) can be displayed on T4 capsid individually. Additionally, T4 phage platform can display antigens *in vitro* by incubating the purified Soc-antigen fusion proteins with purified *Hoc^−^Soc^−^*T4 phage (Tao et al., 2017a), which is critical for the display of conformation-sensitive proteins such as flu HA trimer and HIV gp140 trimer. Due to the absence of post-translational modification pathways in bacteria, phages cannot be used to *in vivo* display antigens that require post-translation modifications, such as glycosylation that is important for structural and conformational integrity of the protein.

As natural protein nanoparticles, phages are able to elicit immune responses (Dabrowska et al., 2014), thus potentially limiting their use when multiple vaccinations are needed. However, with recent progress on phage engineering, as discussed above, this can be minimized. The epitope regions of phage capsid proteins, like any other pathogen, have variable immunogenicity, and the epitope that elicits the most robust immune response is called immunodominant epitope (Akram and Inman, 2012). Therefore, the immunogenicity of phages can be reduced by disrupting the immunodominant epitopes through phage engineering. Second, attachment of polyethylene Glycol (PEG), also known as PEGylation, allows enhanced solubility as well as a reduction in the renal clearance hence, extending time in circulation (Suk et al., 2016).

### Phage Therapies

Phages were used to treat bacterial infections since their discovery in the early 20th century (Lu and Koeris, 2011; Wittebole et al., 2014; Moelling et al., 2018). Although discontinued in Western countries since the discovery of antibiotics in the 1940s (Moelling et al., 2018), the phage therapy was recently suggested by NIH as one of the seven innovative approaches to antimicrobial resistance research (National Institute of Allergy and Infectious Diseases, 2014). About 100 years of clinical usage of phage therapy in some Eastern European countries indicates that it could be a promising approach, particularly now that approaches for discovery of new broad spectrum antibiotics have nearly been exhausted (Moelling et al., 2018). In the past several years, we have isolated many phages from different bacterial hosts and showed their application to treat bacterial infection in animal models (Chen et al., 2018a,b,c, 2019a,b). Here, we are not going to much detail of phage therapy, but discuss the unique properties of phages that make them a promising alternative or supplement of antibiotics to treat bacterial pathogens. Phage engineering provides a rapid strategy to generate phage variants with unique properties, which might accelerate the development of phage therapy. For more detail of clinical application of phage therapy and related regulator obstacles, we encourage the reader to refer to the recent review articles (Kutter et al., 2010; Miedzybrodzki et al., 2012; Carvalho et al., 2017; Expert Round Table on Acceptance and Re-Implementation of Bacteriophage Therapy et al., 2018; Pirnay et al., 2018; Svircev et al., 2018; Kortright et al., 2019).

Unlike antibiotics or other chemical medicines, phages are natural organisms that can replicate in their host bacteria. This makes them an ideal weapon to fight against bacterial infections. In theory, a relatively small number of phages deposited at the site of infection are enough to treat a bacterial infection because of their replication and self-amplification. Once the pathogen was eliminated, phages no longer replicate and can be quickly cleared by the immune system or other mechanisms. For example, when phage MR-10 (10^8^ PFU/ml) was injected into the hind paw of mice pre-infected by *Staphylococcus aureus* (10^6^ CFU/ml), phage titers initially increased on days 1 and 3 but declined on day 5. The time course correlated very well with the clearance of *S. aureus* (Chhibber et al., 2013). Secondly, the phages evolve with the selection condition, which helps them overcome bacterial resistance mechanisms. For instance, bacteria employ restriction-modification (R-M) systems to destroy invading DNA, while keeping the self-DNA safe by methylation of specific sites (Samson et al., 2013; Seed, 2015). However, phages can incorporate base modification systems to keep their genome resistant to the bacterial R-M systems (Samson et al., 2013). For example, T4 phage modifies the cytosines by two modifications, 5-hydroxymethylation and glucosylation, which make it highly resistant to virtually all the restriction endonucleases of *E. coli* (Bryson et al., 2015). Bacterial CRISPR-Cas immune system is another well-studied anti-phage mechanism that protects the host through cleavage of phage DNA (Hille et al., 2018). Phages can evade CRISPR-Cas through either mutation of key nucleotides responsible for CRISPR-Cas complex binding/cleavage (Tao et al., 2018b) or expressing anti-CRISPR proteins (Pawluk et al., 2018). Modulating the availability of the receptors is another common mechanism employed by bacteria to block phage infection (Seed, 2015). However, phages can regain the ability of binding to their receptor by modifying the receptor-binding protein to adsorb to the evolving bacterial populations (Samson et al., 2013). Therefore, the endless coevolution of phages and their host bacteria makes bacteria less resistant to phage therapy than the antibiotic treatment, especially when phage cocktails are used.

Phages have high host specificity. Typically, a single type of phage can only recognize a limited range of bacterial strains (Nobrega et al., 2018). Therefore, they can be used to treat a specific bacterial pathogen without causing damage to the otherwise normal microbial community of the host. However, the narrow host range makes it almost impossible to target all strains within a given species using a single phage type. Thus, phage therapy requires the identification of pathogenic strain followed by the selection of effective phages, which will delay the treatment. However, there are ways to overcome this limitation (De Jonge et al., 2019). First, the host range of phages can be expanded or changed by genetic engineering techniques discussed above to manipulate the receptor-binding proteins. Swapping the receptor-binding protein genes between different types of phages, which have a different host, was able to change host specificity. For instance, replacing the long tail fiber genes of T2 phage with those from phage PP01 shifted the host of T2 from *E. coli*-K12 to *E. coli* O157:H7 (Yoichi et al., 2005). Similarly, replacing the long tail fiber genes with those from phage IP008, which has wide host range (can infect more *E. coli* strains), shifted the host range of T2 phage as that of IP008 (Mahichi et al., 2009). Swapping receptor-binding protein genes between more distant phages could even enable an engineered *E. coli* phage to infect *Klebsiella* bacteria and vice versa (Ando et al., 2015). The host range of phage can also be expanded by incorporation of a heterologous receptor binding domain (Marzari et al., 1997). For instance, filamentous phage fd infects *E. coli* bearing F pili, whereas filamentous IKe infects *E. coli* containing N or I pili. Fusion of the receptor-binding domain of IKe gene 3 protein (pIII) to the N terminus of the fd pIII expanded the host range of fd phage (Marzari et al., 1997). The modified fd phage is able to infect *E. coli* bearing either N or F pili. Strikingly, fd was engineered to infect *V. cholerae* by adding the N-terminal 274 amino acids of pIII from filamentous phage CTXphi, which infects *V. cholerae* by toxin-coregulated pili, to the N terminus of the fd pIII (Heilpern and Waldor, 2003). Secondly, multiple phages targeting different strains can be isolated from natural environment to target more strains. A good example is the recent case of the “San Diego patient,” who was infected with a multi-drug resistant *A. baumannii* strain and recovered after intravenous injection of phage cocktails (Schooley et al., 2017). However, this was no control here to assess if the therapeutic effect was entirely due to treatment with the phage cocktail. After screening more than 100 different phages isolated from environments, 9 phages that can lytic the patient’s *A. baumannii* strain were selected to form three cocktails to treat the infection (Schooley et al., 2017). A number of phages targeting different *Pasteurella multocida* strains have been isolated (Chen et al., 2018b,c, 2019a), and the therapeutic effect of the phage cocktail is under investigation. Alternatively, phage-derived enzymes, such as virion-associated lysins, endolysin, and depolymerase, can be used to lyse bacteria (Maciejewska et al., 2018). For example, we showed that the depolymerase of phage PHB02, when was inoculated intraperitoneally, significantly increased the survival of mice pre-infected with *P. multocida* (Chen et al., 2018c). Although some of phage-derived enzymes also have narrow specificity, they are able to lysis a given bacterial specie other than a single strain (Maciejewska et al., 2018).

The most attractiveness of phages therapy is that it can be used to eliminate drug-resistant bacteria (Viertel et al., 2014). Phages and antibiotics have an intrinsic difference in their mechanisms of killing bacterial pathogens, and thus, there is no cross-resistance to antibiotics and phages. Technically, phages have the same efficiency to lyse antibiotic resistant bacteria as that of the antibiotic sensitive ones. Furthermore, phages and antibiotics can be combined to treat a bacterial infection (Torres-Barcelo and Hochberg, 2016). For instance, Kirby’s study indicated that the combined use of gentamicin and phage (SA5) can be more efficacious than single therapies using either gentamicin or phage SA5 to treat *Staphylococcus aureus*. (Kirby, 2012). Similarly, combining phage LUZ7 and streptomycin decreased the titer of *Pseudomonas aeruginosa* compared to either treatment separately (Torres-Barcelo et al., 2014). This is also true in a diabetic mouse model, in which the hind paw of each mouse was infected with *S. aureus* (Chhibber et al., 2013). The maximum reduction of bacterial titer was obtained when phage MR-10 and linezolid were simultaneously used to treat the infection (Chhibber et al., 2013). The phage-antibiotic combinations not only enhance the eradication of bacteria but also prevent the emergence of resistant variants, compared to treatment with either phage or antibiotic alone. For instance, Verma et al. showed that the combination treatment using ciprofloxacin and phage KPO1K2 can not only eradicate the *Klebsiella pneumoniae* biofilm but also significantly arrest the emergence of resistant variants *in vitro* (Verma et al., 2009). Recent findings from Turner’s group provided one of the mechanisms that phage infection might affect drug resistance of its host bacteria through evolutionary tradeoff between phage resistance and antibiotic resistance (Chan et al., 2016, 2018). They isolated a lytic phage, OMKO1, which infects *Pseudomonas aeruginosa* using the outer membrane porin M (OprM) as a receptor. OprM channel is a part of the antibiotic efflux pump of *P. aeruginosa*. Infection of phage OMKO1 led to selection of OprM mutations that affected its efflux function and restored antibiotic sensitivity of *P. aeruginosa*. Recent studies found that combination treatment using phage and sub-lethal concentrations of certain antibiotics would increase host bacterial production of phages, which was called phage-antibiotic synergy (PAS) phenomenon (Comeau et al., 2007; Kamal and Dennis, 2015). For instance, the production of phage ΦMFP in an uropathogenic *E. coli* strain increased more than sevenfold when 20 ng/ml cefotaxime was added to the medium (Comeau et al., 2007). This is also true in case of phage KS12, which infects *Burkholderia cenocepacia* strain K56–2 (Kamal and Dennis, 2015). The diameter of phage KS12 plaque increased from 1.22 to 2.37 mm when 4X the minimum inhibitory concentration of meropenem was added to the medium (Kamal and Dennis, 2015). *In vivo* experiments also showed that treatment with a combination of phage KS12 and 6 μg/ml meropenem increased the survival of Galleria mellonella larvae pre-infected with 9 LD50 (50% lethal dose) of *B. cenocepacia* K56–2 compared to controls treated with KS12 or antibiotic alone (Kamal and Dennis, 2015).

Other than having direct antimicrobial activity, phages can be easily engineered using genetic engineering approaches to carry genes, proteins, or antimicrobial chemicals to enhance their antimicrobial activity. One good example is the delivery of biofilm-degrading enzyme dispersin B (DspB) using an engineered T7 phage (Lu and Collins, 2007). A biofilm is a structured community of microorganisms producing a polymeric matrix, which might make bacteria resistant to antimicrobial agents such as antibiotics and phages. The engineered phage T7 expressed *DspB* gene of *Actinobacillus actinomycetemcomitans* derived by T7 φ10 promoter, which can be recognized by T7 RNA polymerase, therefore can significantly reduce bacterial count in a single-species *E. coli* biofilm than the T7 phage control did (Lu and Collins, 2007). Similarly, T7 phage was engineered to express a lactonase enzyme that interfered with the quorum sensing, which plays important role on biofilm formation. The resulted T7 phage reduced the biofilm formation by 74.9 and 65.9% at 4 and 8 h post-plating, respectively, compared to no-phage control. However, the wild-type T7 phage reduced only 23.8 and 31.7% at 4 and 8 h, respectively (Pei and Lamas-Samanamud, 2014). Other than delivering genes targeting biofilms, phages were also used to deliver an antibiotic drug or a CRISPR-Cas system (Bikard et al., 2014; Citorik et al., 2014) that is programmed to cleave a specific gene such as the antibiotic resistance genes. For instance, phagemids encoding the CRISPR-Cas9 system, which was programmed to target the *aph-3* kanamycin resistance gene, was packaged in the *Staphylococcal* phage ΦNM1 (Bikard et al., 2014). When *S. aureus* RN4220 cells carrying a kanamycin resistance gene were infected with the recombinant ΦNM1phage, strong inhibition of bacterial growth was observed. Conversely, the ΦNM1phage packaged with non-targeting CRISPR-Cas system did not produce significant inhibition.

Although phage therapy has been used to prevent or treat bacterial infection for almost 100 years particularly in Eastern European counties such as Georgia, it has not been well accepted in Western countries. One reason is the lack of detail information on the early clinical trials or applications. No details on the experiment design or data analysis were provided, although some of the clinical data were available in their internal publications (Kutter et al., 2010). The first placebo-controlled, double-blind clinical trials were published in 2009 targeting to drug-resistant *P. aeruginosa*, which causes chronic otitis (Wright et al., 2009). This study showed encouraging results in that phage treatment resulted in significant clinical improvement compared to placebo controls. Since then, 14 clinical trials have been launched or completely carried out (Expert Round Table on Acceptance and Re-Implementation of Bacteriophage Therapy et al., 2018). However, most of these clinical results have not published yet, which are needed to further evaluate the potential of phages in treating bacterial infections (Expert Round Table on Acceptance and Re-Implementation of Bacteriophage Therapy et al., 2018). For more detail from clinical aspect of phage therapy, we encourage the reader to refer to the recent elegant review articles (Kutter et al., 2010; Miedzybrodzki et al., 2012). Another challenge of phage therapy is government regulatory hurdles especially in Western countries. Phages were classified as drugs and medicinal products in United States and European Union, respectively (Pirnay et al., 2018). Therefore, it requires strict clinical trials and complicated drug approval procedures before reaching market. However, the recent pragmatic phage therapy framework that centers on the “magistral preparation” of tailor-made phage medicines in Belgium might provide a light for future clinical application of phages (Pirnay et al., 2018).

## Conclusions

The emergence of multi-antibiotic resistant bacterial pathogens and their continuing spread in the population brought new urgency to develop alternative strategies to treat bacterial infections. Phage therapy and phage nanoparticle vaccination could be two promising strategies to address this crisis. Successful treatment of the “San Diego patient” and several ongoing phage therapy clinical trials demonstrate the potential of this approach to develop phage antibiotics (Schooley et al., 2017; Expert Round Table on Acceptance and Re-Implementation of Bacteriophage Therapy et al., 2018). Phage VLP vaccines have shown high efficacy in the animal models, and some have already entered clinical trials (Huang et al., 2017; Tao et al., 2018c). There are however limitations to the naturally occurring phages but, fortunately, recent progress in phage genome engineering promises to overcome these limits, such as expanding phage host range to facilitate phage therapy and disrupting the immunodominant epitope of phage capsid to eliminate immune response against phage and, therefore, to generate precise variants against infectious diseases (Nobrega et al., 2015; Bardy et al., 2016; Pires et al., 2016; Kilcher and Loessner, 2019).

## Author Contributions

YC, HB, JD, CC, and PT wrote the manuscript. VR and PT edited the manuscript.

### Conflict of Interest Statement

The authors declare that the research was conducted in the absence of any commercial or financial relationships that could be construed as a potential conflict of interest.
